# Dynamic Velocity Tracking in the Left‐Ventricular Outflow Track Using Four‐Dimensional 4D Flow for the Assessment of Left Ventricular Outflow Track Obstruction

**DOI:** 10.1002/ccr3.70465

**Published:** 2025-04-21

**Authors:** Chris Sawh, Pankaj Garg

**Affiliations:** ^1^ Norfolk and Norwich University Hospitals NHS Foundation Trust Norfolk UK; ^2^ Norwich Medical School University of East Anglia Norfolk UK

**Keywords:** cardiology, cardiovascular disorders, dyspnea, mitral valve, perfusion imaging

## Abstract

Stress 4D flow CMR is a powerful non‐invasive tool for diagnosing and localizing dynamic LVOT obstruction. By quantifying peak velocities and flow patterns under stress, it provides critical insights into hemodynamics, especially when echocardiography is limited, enabling accurate diagnosis and improved clinical decision‐making in complex cases.

A 58‐year‐old woman presented to the cardiology outpatient clinic with progressive shortness of breath. Her medical history included diabetes mellitus, hypertension, and hypercholesterolaemia. Initial electrocardiography showed sinus rhythm with T‐wave inversion in leads I and aVL. Transthoracic echocardiography demonstrated a normal‐sized left ventricle with preserved systolic function; however, acoustic windows were suboptimal due to body habitus. A sigmoidal basal septum was noted, resulting in systolic anterior motion (SAM) of the mitral valve and turbulent flow in the left ventricular outflow tract (LVOT), though quantification was not possible due to alignment challenges. The right ventricle was structurally and functionally normal, and no significant valvular pathology was identified.

To further evaluate cardiac function and investigate myocardial ischaemia, the patient underwent cardiovascular magnetic resonance (CMR) imaging with first‐pass perfusion and 4D flow assessment [1, 2]. Given the echocardiographic suspicion of LVOT obstruction, a tailored protocol incorporating rest and peak‐stress 4D flow CMR was applied. Dynamic velocity tracking of three‐dimensional streamlines enabled precise evaluation of flow patterns and velocities in the LVOT.

At rest, blood flow through the LVOT was smooth, with a peak velocity of 170 cm/s located just below the aortic valve, within normal limits. During adenosine‐induced hyperaemia, first‐pass perfusion imaging revealed myocardial ischaemia in the anterolateral wall (Figure 1a–c). Stress 4D flow CMR demonstrated marked flow acceleration with a high‐velocity jet near the septal bulge, coinciding with a rise in peak velocity to 250 cm/s and increased streamline density (Figure 1d–f). These findings confirmed dynamic LVOT obstruction under stress, with clear spatial localization of flow disturbance.

**FIGURE 1:**
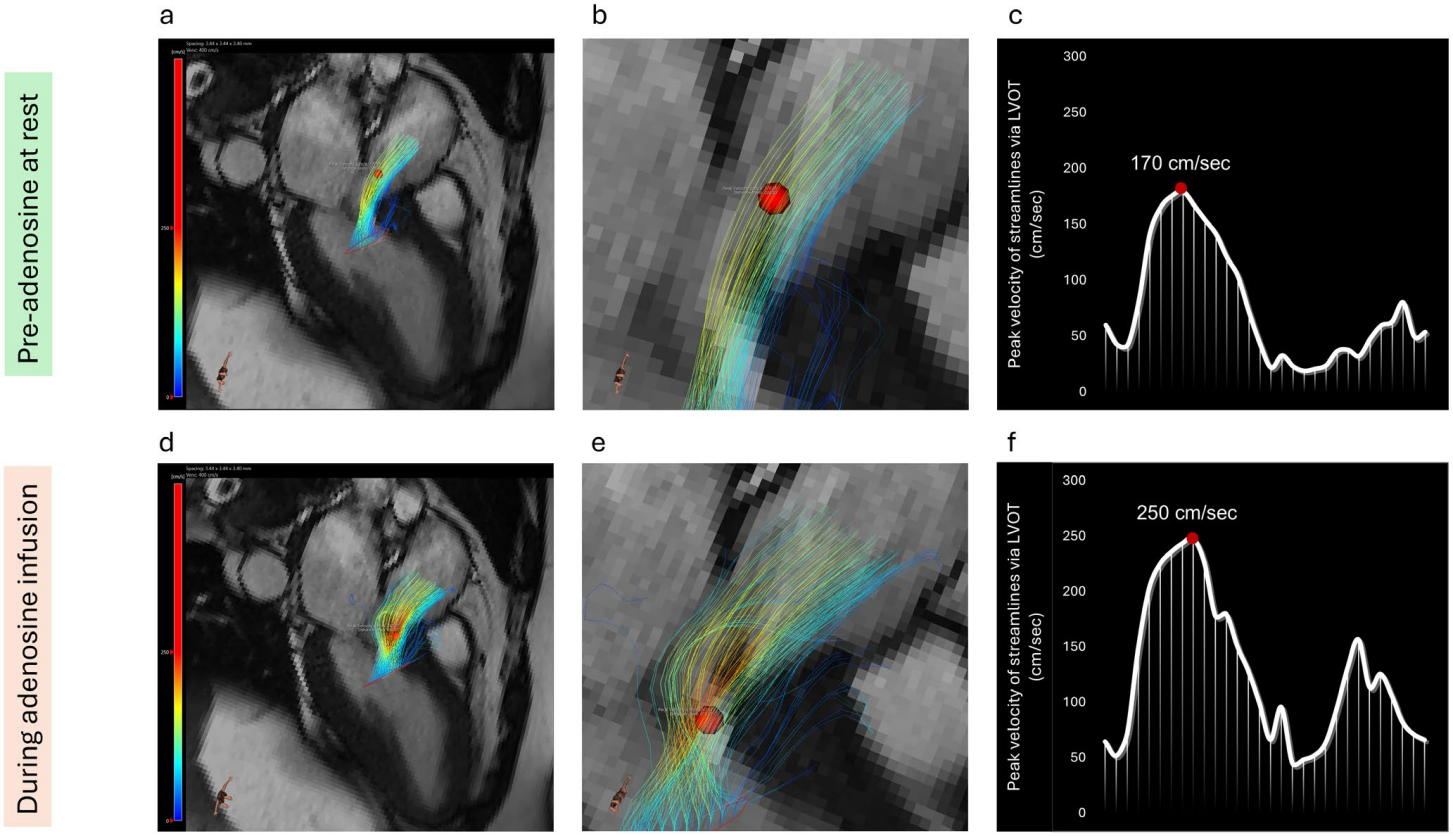
Dynamic 4D Flow CMR Analysis of Peak Velocity in the LVOT to Assess Left Ventricular Outflow Tract Obstruction (LVOTO). Panel (a–c) Pre‐adenosine at rest: 4D flow CMR‐derived 3D streamlines in the LVOT (a) show smooth blood flow with no significant acceleration, confirmed in the zoomed‐in view (b) where the peak velocity (red dot) remains low. The velocity‐time curve (c) indicates a peak velocity of 170 cm/s, within the normal range. Panel (d–f) During adenosine infusion: Under adenosine stress, 3D streamlines (d) show marked flow acceleration and a high‐velocity jet in the LVOT. The zoomed‐in view (e) highlights increased streamline density and convergence at the peak velocity location (red dot). The velocity‐time curve (f) demonstrates a significant rise in peak velocity to 250 cm/s, exceeding the diagnostic threshold for LVOTO.

This case underscores the clinical value of stress 4D flow CMR as a novel, non‐invasive modality for the comprehensive assessment of suspected LVOT obstruction. By dynamically quantifying flow velocities and identifying the precise location of obstruction, this technique provides critical insights into the hemodynamics of flow acceleration and its pathophysiological impact.

The role of CMR imaging in the assessment of cardiovascular diseases is expanding [3]. In particular, 4D flow CMR is increasingly gaining attention as it circumvents issues of routine Doppler assessment, mainly the operator dependence of flow and velocity quantification. In slow flow aortic stenosis (AS), this technique may enhance the precision of stenosis severity assessment by quantifying pressure gradients during increased cardiac output. For LVOT obstruction in hypertrophic cardiomyopathy (HCM), stress 4D flow CMR can reveal the dynamic nature of obstruction under stress conditions, providing critical insights for patient stratification.

1

Stress 4D flow CMR faces some barriers to clinical adoption. The 5‐to‐10‐min acquisition time complicates maintaining consistent physiological stress, particularly during exercise. Although pharmacological stressors mitigate these, ensuring steady stress levels throughout the scan remains difficult. Additionally, the lack of standardized 4D flow sequences across imaging centres and the scarcity of expertise hinder its broader translation into routine practice.

To our knowledge, this is the first reported case utilizing stress 4D flow CMR to diagnose and localize dynamic LVOT obstruction with concurrent evidence of myocardial ischaemia. This approach demonstrates its potential to enhance diagnostic accuracy and guide tailored management strategies in complex cardiomyopathies.

## Author Contributions

**Pankaj Garg:** data curation, formal analysis, methodology, project administration, software, writing – review and editing. **Chris Sawh:** conceptualization, data curation, investigation, methodology, resources, supervision, validation, writing – original draft.

## Consent

The authors confirm that written consent for submission and publication of this case report, including images and associated text, has been obtained from the patient in line with COPE guidance.

## Conflicts of Interest

P.G. has a clinical advisor role with Medis Medical Imaging and Pie Medical Imaging. P.G. consults for Edward Lifesciences and Anteris.

## Data Availability

All data underlying this article are available as part of the article. No additional source data are required.
